# Alternative Therapeutic Intervention for Individuals with Rett Syndrome

**DOI:** 10.1100/tsw.2007.4

**Published:** 2007-05-29

**Authors:** Meir Lotan

**Affiliations:** ^1^ Israeli Rett Center, National Evaluation Team, Chaim Sheba Medical Center, Tel HaShomer, Ramat Gan, Israel; ^2^ Department of Physical Therapy, Academic College of Judea and Samaria, Ariel, Israel

**Keywords:** Rett syndrome, alternative treatment, allied professions

## Abstract

The individual with Rett syndrome (RS) displays an array of challenging difficulties in all areas of daily living. Since there is no cure for the disorder at this moment, parents of the individual with Rett search for different interventional modalities that will improve the condition and quality of life for their child. During the last few years, many individuals with RS have experienced different kinds of interventions. This paper presents these methods with relevant case stories for others to share the possibilities. This paper reviews the following interventions: animal-assisted therapy, such as dolphin therapy and dog-assisted therapy; auditory integration training; hyperbaric chamber; manual therapy, such as acupuncture/acupressure, aromatherapy, craniosacral therapy, Mayo facial release, Treager massage, chiropractor, and Reiki; mental modification techniques, such as Lovas and cognitive rehabilitation; motoric interventions, such as advanced biomechanical rehabilitation, patterning/Doman-DeLacato approach, and yoga. The present paper is not a recommendation for any of the above-mentioned techniques, but merely a review of different interventions available for the inquisitive parent of the individual with RS.

### INTRODUCTION

Rett syndrome (RS) is a neurological disorder resulting from an X-linked dominant mutation[1], affecting mainly females and found in a variety of racial and ethnic groups worldwide[2]. RS is a frequent cause of neurological dysfunction in females, accounting for the second-most common cause for multiple disability among females, after Down's syndrome[3,4]. Due to the chronic nature of RS and to the fact that the individual with RS displays an array of challenging difficulties affecting all areas of daily living[3], it is not surprising that putative cures surface.

Since there is no cure for the disorder at this moment, parents of the individual with Rett are in a search for different interventional techniques that will improve the child's condition and quality of life. It is common with these alternative methods that some initial reports relate to an individual child or a handful of cases. Such reports are difficult to interpret as the many aspects of the presented intervention approach may be vague or otherwise highly doubtful. Such reports usually are not followed by more rigorous and controlled studies in the peer-reviewed scientific literature.

In many cases, alternative intervention suggests "nothing but" a short, positive, and relaxing experience for the child with RS. Due to the turmoil nature of this disorder, attributing overactive autonomic system (placing the individual with RS in a state of constant arousal)[5], and highly developed emotional being of individuals with RS[6], it could be just what they need.

Parents of the individual with RS should be cautious of different unconventional interventional possibilities and make well-informed decisions (as best as possible regarding the vagueness of the approach at hand) regarding the use of alternative treatments for their daughter. In this review, a treatment approach posing some risk to the client with RS has been noted in text boxes and should be carefully taken into consideration (see Box 1).

**Wards of warning:**

- There is a vast selection of available interventions; shop for them cautiously.
- Intervention involving termination of all other co-existing interventions for the individual with RS for the duration of treatment should be cautiously reconsidered and preferably avoided.
- After deciding on a therapy program, a time limit for its duration with pre-excepted results should be set.
- If by the end of a pre-determined set of intervention sessions a therapeutic result is not visible, continuation of the program should be reconsidered.
- A quick and constant change between intervention methods should be avoided.

**Box 1**

During the last few years, many individuals with RS have experienced different kinds of interventional techniques. This review will present these methods through a short description of each one, followed by an appended case story of an individual with RS that has experienced it. When reading through the case stories, the reader should bear in mind the enormous phenotypic versatility presented by individuals with RS, suggesting that in case of a different child, the results of the same intervention might be dissimilar or even of an opposite nature.

This review displays the following interventions according to alphabetical order:

- Animal-Assisted Therapy
  - Dog-Assisted Therapy
  - Dolphin Therapy
- Auditory Integration Training (AIT)
- Hyperbaric Chamber.
- Manual Therapy
  - Acupuncture/Acupressure
  - Aromatherapy
  - Chiropractor
  - Craniosacral Therapy
  - Mayo Facial Release
  - Reiki

- Treager Massage
- Mental Modification Techniques
    - Cognitive Rehabilitation
    - Lovaas
- Motoric Interventions
    - Advanced Biomechanical Rehabilitation (ABR)
    - Patterning/Doman-Delacato Approach
- Yoga

It is reiterated that the present review is not a promotion for any of the above-mentioned techniques, merely a presentation of different interventions available for the inquisitive parent of the individual with RS.

### Animal-Assisted Therapy

Animal-assisted therapy (AAT) is a goal-directed intervention in which an animal that meets specific criteria is an integral part of a treatment that facilitates the healing process and rehabilitation of clients with acute or chronic disease[7,8]. Therapeutic horseback riding is also a part of AAT, which has been mentioned elsewhere and therefore will not be addressed within the scope of the present article. AAT interventional techniques that have been experienced by individuals with RS are dog-assisted therapy and dolphin therapy.

#### *Dog-Assisted Therapy*

Results showed that children with pervasive developmental disorders (PDD) exhibited a more playful mood, were more focused and more aware of their social environments, when in the presence of a therapeutic dog[8]. Individuals with RS are extremely fond of animals. Different reports suggested that animals, mainly dogs, have been a powerful motivating factor for individuals with RS. When the family dog would escort the child with RS to physical therapy sessions, the child would cooperate and more willingly experience different and far more challenging activities beyond their usual level of cooperation. It was also reported that dogs were used for reducing stereotypical hand movements due to the individual’s motivation to pat the dogs[9].

##### Story of Claire

Claire loves dogs. The only three words she spoke in her life were “cookie” (briefly when she was six) and “dog” and “again” when she was two. “Dog” was her first word, spoken the first time she saw a friend's collie, and again later when she saw a neighbor's dog. She is attracted to large dogs and is motivated to touch their faces. Her love for dogs is strong enough to get her to actually use her hands to throw a ball to Paula's dog (see Pictures 1 and 2)[10].

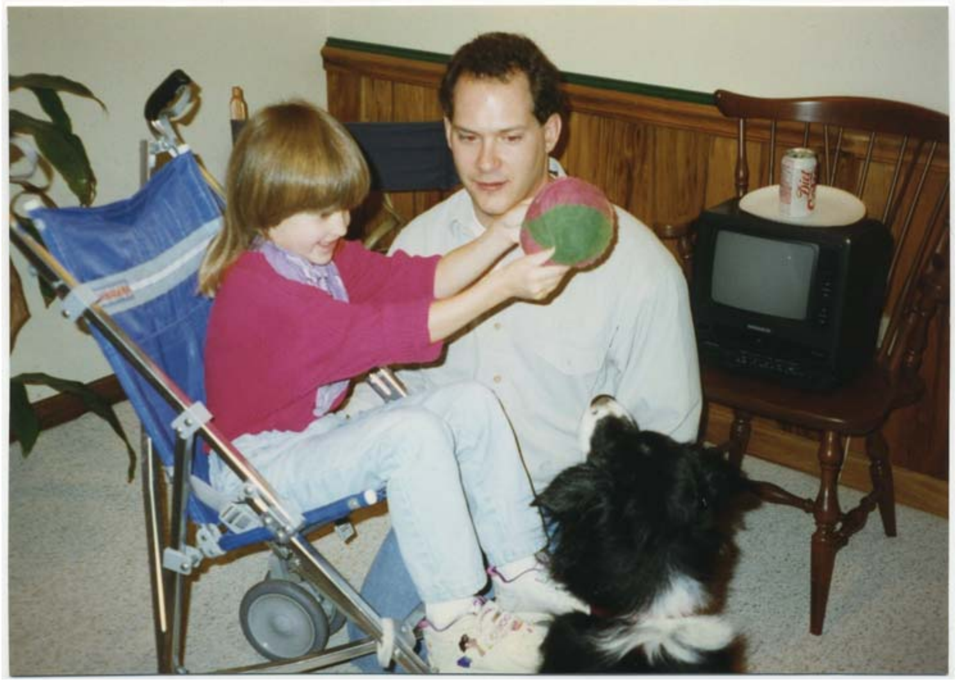

**PICTURE 1**

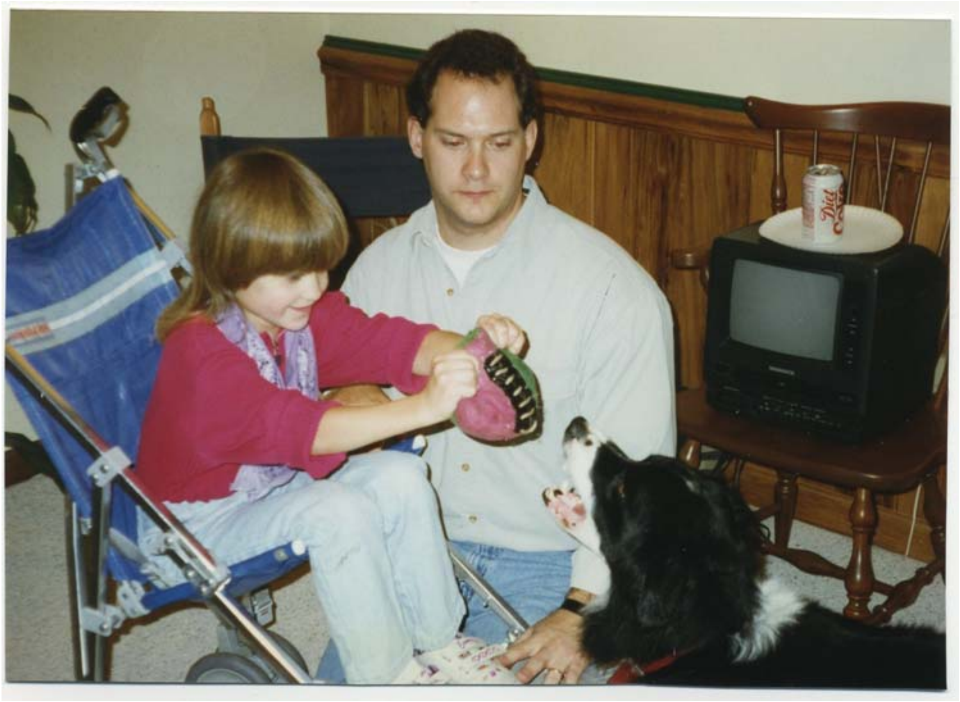

**PICTURE 2**

### *Dolphin Therapy*

Dolphin therapy for individuals with developmental disabilities is well documented[11] and is thought to originate from the dolphins' special capabilities of enhancing healing potential in people[12] as well as from the fact that "dolphins are such gentle and perceptive animals and children really respond to them." They attract attention, stimulate awareness and interest, as well as provoke excitement and motivation[13], therefore, they are extremely suitable for individuals with RS. Furthermore, the combination of the environment, the movement of the water, the texture of the dolphin's skin, and the overall experience is so engaging that the child's ability to interact and learn is increased[14]. All families that have reported this form of intervention have mentioned that it was a unique and extremely positive experience both for the child with RS as well as for themselves. One drawback was the fact that it was extremely expensive, usually involving the relocation of the whole family for a short and defined period[14,15,16]. Due to the costs involved in this type of intervention, it is usually a single experience.

#### Story of Emily

The intervention that Emily went to was an intense 10 days with 40 min of therapy a day. In Emily's case, she was matched with a therapist to suit her needs. The first time she went, she had a physiotherapist who worked with her on the dockside in the sunshine. On Emily's second trip, a speech therapist massaged her mouth, face, and neck to encourage Emily to talk. Sara, Emily's mother, reported that "Emily had to complete a task, like pointing to 'yes' or 'no' cards in response to a question, and the reward was swimming with the dolphin (see Picture 3). She also sat on the dockside and the dolphin came and kissed her feet, which was lovely." "The most amazing thing was watching Emily being pushed along by the dolphin as she held on to a float. She couldn't talk, but was really excited and made lots of vocal noises. I felt really emotional." Sara also thought it was really worth it. "Before dolphin therapy, Emily was trapped in a world of her own, crying a great deal, but since we've returned, she has been noticeably brighter, more conscious, and she's making more eye contact." "The reaction to the therapy was initially impressive. When Emily got back to school, they could not get over the difference, she just seemed so bright. Today Emily is more able to communicate and occasionally laughs and giggles. We play 'ready steady go!' where she runs from one side of the room to the other. She would never have been able to do that before. Sometimes she really looks at my husband when he is talking and we are sure she knows what he is saying." However, the results did not last. "It did seem to last a while" says Sara, "but after about 3 months I saw her regressing"[13,14].

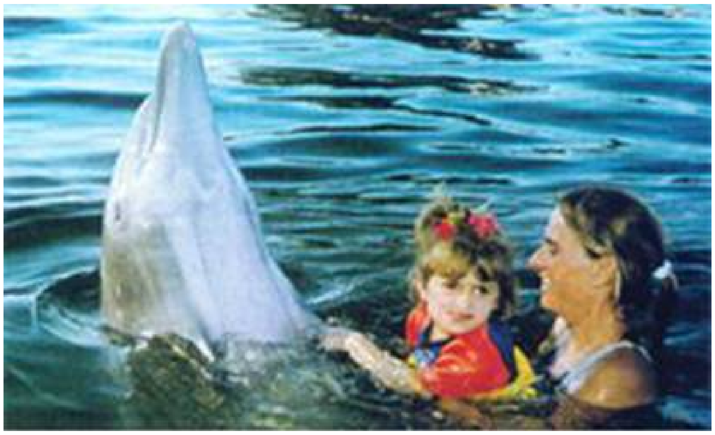

**PICTURE 3.**

### Auditory Integration Training (AIT)

AIT was first developed in France in 1982[17] by Berard and later translated to English[18]. The initiator of AIT postulated that abnormal sensitivity or insensitivity to certain frequencies of sound waves, regardless of overall hearing ability, was associated with a range of behaviors and learning problems[18]. The declared aim of AIT was to correct poor integration between the sensory modalities. Through presentation of special sounds and music over several office sessions, treatment stimulates the brain in a designed manner, as to integrate sensory functioning, thus lessening distractibility and hyperactivity. AIT involves 10 h of listening to electronically modified music delivered by headphones during two half-hour daily sessions over the course of 10 days. The AIT device uses filtering to dampen peak frequencies to which the individual is hypersensitive and delivers sounds modulated by random dampening of high and low frequencies and intensities[18].

This method has been presented to a variety of disorders, including autism, depression, hyperactivity, and learning difficulties. Personal accounts demonstrate dramatic improvements in some individuals. Nevertheless, no adequate scientific research presently exists to classify AIT as a useful treatment for the majority of clients[19], but the treatment does show promise for some individuals. It is important that the individual with RS be prescreened in order to detect whether or not she has the particular pattern that is most likely to benefit from AIT.

#### Story of Annette

Annette, age 3, from Canada, received 10 weekly sessions of AIT. According to her parents, she slept throughout the sessions. No noticeable changes were observed as result of her exposure to this intervention program[20].

### Hyperbaric Chamber

Hyperbaric chamber oxygen therapy (HBOT) is the therapeutic administration of 100% oxygen at environmental pressures greater than 1 atmosphere absolute (ATA). This involves placing the patient in an airtight vessel, increasing the pressure within that vessel, and administering 100% oxygen for respiration. In this manner, it is possible to deliver a greatly increased partial pressure of oxygen to the tissues. Typical treatments involve pressurization to between 1.5 and 3.0 ATA for periods between 60 and 120 min, once or more daily. Since the 1960s, there have been reports of HBOT improving the outcome following brain trauma[21]. Administration of HBOT is based on the observation that hypoxia following closed head trauma is an integral part of the secondary brain injury mechanism. Hypoxic neurons performing anaerobic metabolism result in acidosis and an unsustainable reduction in cellular metabolic reserve[22]. As the hypoxic situation persists, the neurons lose their ability to maintain ionic homeostasis, and free oxygen radicals accumulate and degrade cell membranes[23,24]. Eventually, irreversible changes result in unavoidable cell death. This gives some basis to the assertion that a form of therapy designed to increase oxygen availability may improve long-term outcome of neurological damage. The basic assumption behind the use of HBOT is its ability to compensate for decreased blood flow by increasing the oxygen content of plasma and body tissues and can even normalize oxygen levels in ischemic tissue[25].

Since HBOT has been found to improve blood flow in chronic neurological patients[26], this type of intervention has been tried during recent years by different populations, such as children with cerebral palsy (CP)[27] and has been reported to show favorable results in 129 of the total of 139 (93%) pediatric patients treated with HBOT[28]. This type of treatment was found to improve symptoms in autistic individuals[25]. The use of HBOT may also cause negative outcomes (see box #2).

**Caution:**
**1. Since oxygen in high doses is potentially toxic to normally perfuse tissue, and the brain is a particularly at risk organ [30], it is appropriate to postulate that in some patients, HBOT may cause more harm through the action of increased free oxygen radical damage than good through the restoration of aerobic metabolism [31].**
**2. Treatments using HBOT have been known to elicit epileptic seizures**
**3. There are anecdotal findings of the fact that HBOT intervention has caused damage to users with CP shortly after hyperbaric oxygen therapy [32].**

**Box 2**

### Story of Abbey

Abbey is almost 6 years old. She started using the HBOT 3 years ago. Initially, one round of 40 "dives" was made in a big multiplaces chamber at a hyperbaric center, however, 6 months later, her family purchased a portable chamber for home use (see Picture 4). The portable chamber used a lower pressure than those used at HBOT centers. Her mother stated that after having the first few sessions, she seemed to "wake up" by the HBOT, and when they would stand Abbey up, she would have much better balance instead of just flopping over. She also said that when Abbey was regularly in her chamber, she had more energy, was more alert, more communicative, and had fewer illnesses and digestive problems. Her mother sums up the HBOT by saying: "it's a nice, adjunctive therapy that helps everything else we do to be more effective"[29].

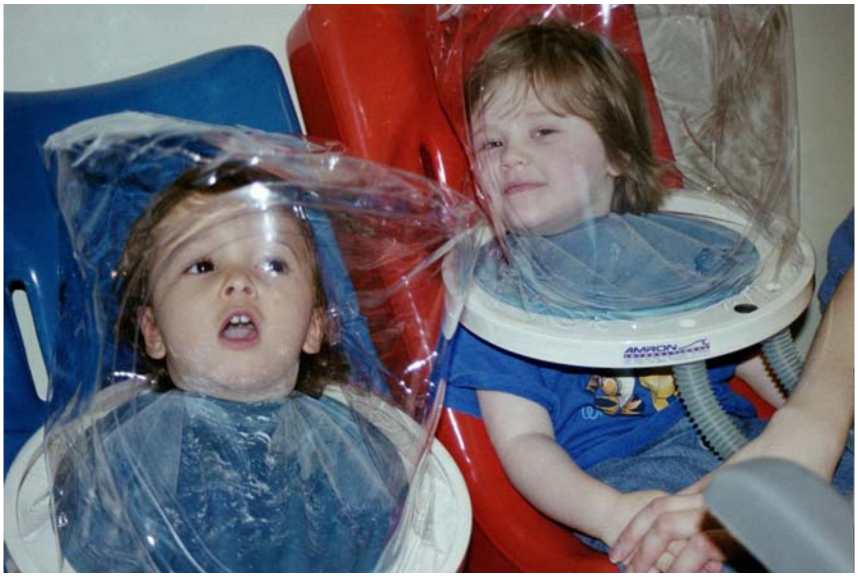

**PICTURE 4**.

### Manual Therapy

#### *Acupuncture/Acupressure*

Acupuncture and acupressure are disciplines extracted from a complex heritage of Chinese medicine. The original authors of Chinese medicine regarded the human body as a microcosmic reflection of the universe and considered the physician's role that of maintaining the body's harmonious balance, both internally and in relation to the external environment. The treatment is executed according to descriptors attached to elemental qualities (wood, fire, earth, metal, and water), and by the functional influences traditionally associated with each of the internal organs. The classical anatomy of acupuncture consists of energy channels (meridians) traversing the body. The principal energy pathways are named for organs whose realms of influence are expanded from their conventional biomedical physiology to include functional, energetic, and metaphorical qualities. Acupuncture involves the therapeutic insertion of solid needles in various combinations and patterns in the body. The adaptability of classical and hybrid acupuncture approaches in Western medical environments is the key to their clinical success and popular appeal[33].

Acupressure is an ancient healing art developed in Asia over 5,000 years ago, using the fingers to press key points on the surface of the skin to stimulate the body's natural self-curative abilities. When these acupressure points are pressed, they release muscular tension and promote the circulation of blood and the body's life force energy to aid healing. Acupuncture and acupressure use the same points and meridians, but acupuncture employs needles, while acupressure uses gentle, but firm, pressure and integrates bodywork therapies, therapeutic touch, somatic work, healing imagery, energy psychology, and massage therapy techniques[34].

Research findings regarding acupuncture: There is agreement that acupuncture appears to be effective for postoperative dental pain, postoperative nausea and vomiting, and chemotherapy-related nausea and vomiting. Positive, yet sometimes confusing, results have been found regarding migraine, low back pain, and temporomandibular disorders, fibromyalgia, osteoarthritis of the knee, and tennis elbow. For conditions such as chronic pain, neck pain, and asthma, the evidence is considered inconclusive and difficult to interpret[35].

Benefits of using acupressure include the relief of pain, balancing of the body, and maintaining of good health. Acupressure's healing touch reduces tension and hypertension[36], increases circulation, and enables the body to relax deeply. By relieving stress, acupressure therapy strengthened resistance to disease and promoted wellness[34,37].

##### Story of Annette

Annett, a 14-year-old child diagnosed with RS. She had received a combination of acupressure, energy healing, and reflexology on a weekly basis for more than a year, since she was 12 years old. Her mother reported that these interventions made her relax during the session, but on the other hand, made her "very upset at times, broke out in hives during some treatments." The treatments did not show any lasting changes[38] (see box #3).

**Reviews have concluded that while not free from serious adverse events (rare they may be), acupuncture is a relatively safe procedure**

**Box 3**

### *Aromatherapy*

Massage is defined as manipulation of the soft tissues of the body performed by the hands of the facilitator for the purpose of producing effects in the vascular, muscular, and nervous systems of the body. Aromatherapy massage uses nonoily, pure volatile portions of aromatic plant products, normally extracted by distillation, as essential oils, for therapeutic or medical purposes. It is the chemical composition of each essential oil that gives the oil its unique therapeutic qualities. In the U.K., the use of essential oils topically is now being employed by some health care professionals as a potential means of enhancing patients' well-being[39]. The use of aromatherapy among the pediatric population is rapidly growing[40]. Essential oils encompass over 60 kinds of herbal oils[41], which are used to ease aching, pain, and injury[42,43]. Aromatherapy also acts on the central nervous system, relieving depression and anxiety, reducing stress, relaxing, sedating or stimulating, and restoring both physical and emotional well-being[44,45,46], improving night sleep[47], and enhancing the immunological system[48].

#### Case Story

The literature reports one child with RS who received aromatherapy. The intended goal of the intervention was to reduce stereotypical hand movements and improve blood circulation to the feet. According to the report, therapy was a success and therapeutic goals achieved[49].

### *Chiropractor*

Chiropractors diagnose and treat patients whose health problems are associated with the body's muscular, nervous, and skeletal systems, especially the spine. Chiropractors believe that interference with these systems impairs the normal functions of the body and lowers its resistance to disease. They also hold that spinal or vertebral dysfunction alters many important body functions by affecting the nervous system, and that skeletal imbalance through joint or articular dysfunction, especially in the spine, can cause pain.

The chiropractic approach to health care is holistic, stressing overall health and wellness. It recognizes that many factors affect health, including exercise, diet, rest, environment, and heredity. In cases in which difficulties can be traced to the involvement of musculoskeletal structures, chiropractors manually adjust the spinal column. Some chiropractors might use water, light, massage, ultrasound, electric acupuncture, and/or heat therapy. Chiropractors counsel patients about wellness through concepts such as nutrition, exercise, changes in lifestyle, and stress management, but do not prescribe drugs or perform surgery[50].

#### Story of Annette

Annette, a 14-year-old individual with RS, from Canada, experienced weekly chiropractic interventional sessions for a period of 2 months when she was 4 years old. The sessions themselves seemed to relax Annette, but no lasting changes were observed as a result of this intervention[51].

### *Craniosacral Therapy*

Craniosacral therapy (CST) is a form of touch therapy in which the bones of the skull are adjusted along with subtle adjustments of the spine, all the way to the sacrum[52]. The basic concept behind CST is that any stresses, strains, tensions, or traumas that have been "stored" in the body will restrict the cerebrospinal fluid circulation, thus harming the body's functioning and may give rise to problems over time. The effects of these stagnation areas may be both physical (such as back pain, migraine, or digestive disorders) and emotional (such as anxiety or depression). Craniosacral therapists are trained to feel these subtle “blocks” in the body and can use their hands to provide the body with an opportunity to let go of its restrictive pattern and return to an easier mode of functioning[53].

#### Case Stories

Allie, is a 7-year-old child with RS who had tried CST. According to her mother, the therapy did not appear to have much of an effect[54]. On the other hand, this type of intervention seemed to reduce teeth grinding in Lauren's case, and her mother, Cathy, recommended it as a good technique for parents seeking some relaxation/recharging time[55] (see box #4).

**Caution: The theory behind cranio-sacral therapy has no anatomical or physiological basis. The intervention purportedly benefits patients with a wide range of diagnoses, and its proponents do not claim any adverse effects while published studies do not provide supporting evidence [56].**

**Box 4**

### *Myofascial Release*

In medical literature, the term myofascial was first used by Janet G. Travell, MD in the 1940s, who referred to musculoskeletal pain syndromes and trigger points. In 1976, Travell began using the term “myofascial trigger point” and published in 1983 the famous reference “Myofascial Pain and Dysfunction: The Trigger Point Manual”. Some practitioners use the term “myofascial therapy” or “myofascial trigger point therapy”, referring to the treatment of trigger points, which is usually in a medical-clinical sense.

The term myofascial release also refers to soft tissue manipulation techniques. It has been loosely used for different manual therapy, applying soft tissue manipulation work (connective tissue massage, soft tissue mobilization, Rolfing, strain-counterstrain, etc). Myofascial release refers to the manual massage technique of stretching the fascia and releasing bonds between fascia and ligaments, muscles and bones, with the goal of eliminating pain, increasing range of motion, and balancing the body. Fascia is located between the skin and the underlying structure of muscles and bones, and is a seamless web of connective tissue that covers and connects the muscles, organs, and skeletal structures in our body. Muscle and fascia are united, forming the myofascia system.

Injuries, stress, inflammation, trauma, and poor posture can cause restriction to fascia. Since fascia is an interconnected web, the restriction or tightness of the fascia at one place, in time, can spread to other places in the body. The goal of myofascial release is to release fascia restriction and restore its tissue health[57].

#### Story of Corinne

Corinne, a 10-year-old child with RS, had received myofascial release techniques from her physical therapist. Since Corinne had developed some functional scoliosis and at some point, it seemed to have worsened (expressed by the fact her shoulders and back got extremely tight), myofacial techniques were implemented. These techniques seemed to loosen things up considerably and the situation was corrected[58].

#### *Reiki*

Reiki (sometimes spelled "Reichi") is an Asian theory that assumes that humans are energy and that by manipulating this energy with the hands, healing occurs[59]. It is a treatment used by individuals as an alternative or complement to Western medical treatment[60]. Several well-designed studies to date show significant outcomes for conditions such as wound healing and reduction of pain and anxiety[61]. It is also suggested that Energy Healing (EH) may have positive effects on various orthopedic conditions, including muscle and connective tissue problems[61] (see box #5).

##### Story of Annette

Annette, a 14-year-old individual with RS, from Canada. When she was 5 years of age, Annette tried a couple of sessions of Reiki. No lasting changes have been documented[62].

**Energy healing (such as Reiki) is an adjunctive treatment which is noninvasive and poses little downside risk to patients [61].**

Box 5

#### *Treager Massage*

This therapy was developed by boxing trainer Milton Treager, MD, and uses gentle, rhythmic movements to relieve tension, ease movement (especially in joints), and induce relaxation. Compression, elongation, and light bouncing as well as rocking motions are involved[63].

##### Case Story

In one case story presented to the author[64], the parents reported that such a technique, when regularly applied to their child with RS, was relaxing and improved mobility of the individual with RS, despite extremely high muscle tone (spasticity).

### Mental Modification Techniques

#### *Cognitive Rehabilitation*

The goal of cognitive rehabilitation is to achieve the most independent or highest level of functioning. Treatment is based on individualized goals that take into consideration the patient's current pattern of strengths and weaknesses. Cognitive rehabilitation treatment goals include:

- Relearning of targeted mental abilities
- Strengthening of intact functions
- Relearning of social interaction skills
- Substitution of lost functions with new skills
- Optimizing control over the emotional aspects of an individual's functioning, including management of impulsivity and anger

After an initial individual diagnostic interview and discussion of the patient's goals, medical records, and neuropsychological evaluation, results are reviewed. A cognitive rehabilitation plan is then developed trough a collaborative effort. The achievement of goals may include a variety of techniques and tools:

- Auditory and/or visual attention-related tasks
- Memory compensation training, i.e., working around memory problems
- Use of adaptive devices to compensate for deficit abilities and adaptive approaches to targeted tasks
- EEG biofeedback (Neuro-feedback)[65].

Systematic review of the literature from 1998 through 2002 suggested substantial evidence to support cognitive rehabilitation therapies in improving areas such as attention deficits, training for apraxia, functional communication deficits[66]. Cognitive rehabilitation was applied to individuals with various cerebral dysfunctions, including those with developmental disabilities[67].

#### Case Story

A single report was received by a therapist who had worked with a child with atypical RS. He claimed that through the use of cognitive rehabilitation techniques, he had managed to achieve improvements in attention skills and motivation. The therapist strongly recommended the approach for individuals with RS[68].

### *Lovaas Technique/Applied Behavioral Analysis (ABA)*

Applied behavioral analysis (ABA) is the practice of reinforcement of behavior, which leads to the acquisition of new skills. The essence of the ABA is that for many children, the excesses and deficits of their condition result largely from a learning blockage, which can be overcome by intensive teaching. Typically developing children learn without our mediation, or the environment they are born into provides the right conditions to learn language, play, and social skills. Children with PDD (such as individuals with RS) learn a great deal less from the environment. They are often capable of learning, but it takes a much more structured and adapted environment, one where conditions are optimized for acquiring the same skills that typical children learn naturally. ABA is all about setting up rules for the environment, which enable our kids to learn. Conversely, any new behavior that is never rewarded is likely to die out after a while (from lack of motivation to repeat it). In addition, a behavior that results in something unpleasant (an aversive) is even less likely to be repeated. These are the basics of behavioral learning theory[69]. This approach is usually applied for individuals with autism spectrum disorders, although there have been reports regarding the use of this approach for individuals with RS. On the surface, the basic approach would not seem to fit the fragile, highly emotional nature of the individual with RS. Nevertheless, the newer version of ABA, termed gentle teaching (GT), stems from the use of unconditional value of the client and the disuse of averse reinforcers[70]. In this form of ABA, “caregivers are encouraged to increase the level of compassion and warmth”[71]. As a response to a question posed by parents, Vicky Slonims a clinical advisor to the Rett syndrome association in the U.K., responded that she believed “it would be necessary to make significant modifications to the curriculum and teaching style to meet the needs of a person with Rett syndrome”[72]. Some reports regarding the use of ABA with individuals with RS were extremely positive regarding the acquisition of skills and the enhancement of the individual involvement in daily situations[73,74].

#### Story of Kelsey

Kelsey received discrete trial therapy (DTT), based on the principles of ABA for 3 years. “Although Kelsey had not progressed as quickly as some autistic children we have seen progress, she definitely made meaningful and significant progress for herself, and that is what counts.” “I believe all our hard work has actually managed to remediate some of the terrible effects of this disorder such as the loss of purposeful hand skills” said her mother[73].

### Motoric Intervention Techniques

#### *Advanced Biomechanical Rehabilitation (ABR)*

ABR is a unique, biomechanically based rehabilitation approach for children and young adults with brain injury, which brings predictable recovery of musculoskeletal structure and motor functions. It is a hands-on method performed by the parents, who learn the ABR technique and receive individual prescription of applications from the ABR professional staff. ABR is a method that rebuilds even the most severely distorted musculoskeletal structure. The cornerstones of the ABR philosophy are fundamental biomechanical principles of the human body's growth and development[75].

#### Story of Abbey

Abbey received ABR for 2.5 years and her mother reported that the changes she has achieved in her musculoskeletal structure remained, even when the program was interrupted[76] (see box #6).

**Caution: ABR is a completely new technique and the theory behind it has no anatomical or physiological proven basis. The intervention benefits children with a wide array of brain injuries, and there are no!! Published studies to provide supporting evidence for this intervention technique.**

**Box 6**

#### *Patterning/Doman-Delacato Approach*

In the 1960s, a new approach to rehabilitative intervention was initiated by Glen Doman, a physical therapist, and Carl Delacato, an educator, known as the “Doman-Delacato treatment” or “patterning”. The Doman-Delacato treatment of patterning is based on theories of how ontogeny recapitulates phylogeny, which date back to the 1920–30s. Those theories are not supported by present knowledge of the development of the central nervous system in humans[77]. Their basic idea was that one can improve function by stimulating specific sensory inputs, thereby eliciting specific motor output patterns[78]. Accomplishment of brain development is achieved by stimulating the brain through movement of the body; for instance, if the children could not walk well, they were given the opportunity to learn crawling through many hours of therapy each day. The claim of Doman and Delacato was that the majority of disabilities for individuals with mental retardation, learning problems, and behavioral disorders were caused by poor neurological organization[79]. According to most researchers, studies of patterning have been negative and its use is not recommended[27]. In fact, the extreme financial and emotional burden of such an intensive intervention program applied on the family of the client with developmental disabilities have caused several organizations to issue cautionary statements regarding the treatment's claim (see box #7). Despite this statement, the patterning approach has been tried by individuals with RS.

**Warning:**
Patterning has little effect on functional skills and was found inappropriate for children with motor disabilities [80].

**Box 7**

### Case Story

A very positive report was sent by Jane George, who reported significant improvement to her daughter's functional abilities, especially to crawl on all fours and achieve independent walking. She also recommended the intellectual stimulation. She summed up the experience by saying: I think that the Doman therapy helped my daughter "get the most out of life for many many years"[81].

Heather's mom reported in further detail and with less enthusiasm: Heather, an individual with RS, had experienced the Doman-Delacato intervention. According to her mother, the most favorable training component of the method was the overhead ladder. Training with this element enabled Heather to achieve independent walking for short distances. Heather enjoyed the vestibular part of the program (intended for stimulating the vestibular system). The part of the program called masking (the placing of a plastic mask over the face) dramatically increased her hyperventilation and apneas. No change was observed due to the patterning training in the crawl box. No change was observed also during her participation in the intelligence program. Janet (Heather's mom) suggested to use caution when deciding on participation in such a program, carefully selecting the different parts of the program in accordance to the needs and preferences of the child[82].

## Yoga

Yoga is a traditional Indian culture and way of life, which gives the practitioner a healthy body and a sound mind, and is believed to alleviate stress and induce relaxation[83]. The commonly performed yoga practices include breathing exercises (pranayama), postures (asanas), devotional sessions, and meditation (dhyana). Many branches of yoga have been described such as hatha yoga, karma yoga, bhakti yoga, and raja yoga[84].

There are several reports regarding the use of yoga in intellectual disability, rehabilitation of myocardial infarct patients, hypertension, and other medical disorders. The effect of yoga on the electroencephalogram and the autonomic nervous system has also been studied[85]. Stress is considered an important precipitating factor for seizures and the efficacy of yoga in stress reduction has been documented[86,87]. It is therefore believed that yoga could be considered as a possible therapeutic option or at least a reducing agent for epilepsy, in view of its nonpharmacological nature, minimal adverse effects, and international acceptance. Yoga has been reported as a physical activity available to individuals with severe disabilities[88].

### Case Story

Jeanne reported that her daughter with RS had received yoga intervention by a trained pediatric special-needs practitioner. She conveyed that the intervention was "great for relaxation and flexibility"[89].

### SUMMARY

This review has presented a minuscule sample of alternative intervention approaches, which were experienced by individuals with RS. The therapist or parent that seeks to expend the child's experience and to explore new intervention options will find some possibilities within the scope of the present article. The cornerstone for implementing a new and nontraditional approach is, of course, the fact that no harm will be afflicted on the child and her family. It is reiterated that every new therapeutic path should be considered and discussed with individuals familiar with the novel intended method and applied only with specific, measurable goals that may be realistically reached within a preset duration. There is a need for larger samples and detailed research before it can be considered that a given treatment has any benefit for persons with RS.

## References

1. Amir, R.E., Van Den Veyver, I.B., Schultz, R., Malicki, D.M., Tran, C.Q., Dahle, E.J., Philippi, A., Timer, L., Percy, A.K., Motil, K.J., Lichtarge, O., Smith, E.O., Glaze, D.G., and Zoghbi, H.Y. (2000) Influence of mutation type and X chromosome inactivation on Rett syndrome phenotypes. *Ann. Neurol.* **47,** 670–679.
2. Hagberg, B., Aicardi, J., Dias, K., and Ramos, O. (1983) A progressive syndrome of autism, dementia, ataxia, and loss of purposeful hand use in girls: Rett syndrome: report of 35 cases. *Ann. Neurol.* **14,** 471–479.
3. Hagberg, B., Ed. (1993). *Rett Syndrome: Clinical and Biological Aspects*. Mac Keith Press, London.
4. Ellaway, C. and Christodoulou, J. (2001) Rett syndrome: clinical characteristics and recent genetic advances. *Disabil. Rehabil.* **23,** 98–106.
5. Kerr, A.M. and Witt Engerstrom, I., Eds. (2001) *Rett Disorder and the Developing Brain*. Oxford University Press.
6. Elefant, C. (2005) Emotional/Musical Communication of Children with RS. A lecture at an annual coference on RS. Sheba Hospital, Ramat-Gan, Israel. July.
7. Rothe, E.Q., Vega, B.J., Torres, R.M., Soler, S.M.C, and Pazos, R.M.M. (2005) From kids and horses: equine facilitated psychotherapy for children. *Int. J. Clin. Health Psychol.* **5(2),** 373–383.
8. Martin, F. and Farnum, J. (2002) Animal-assisted therapy for children with pervasive developmental disorders. *West. J. Nurs. Res*. **24(6),** 657–670.
9. Levasseur, K.M. (1996) Pet Therapy. IRSA Newsletter. Spring.
10. Personal communication (XVI): Contact Bundlings.com. Received on July 1, 2006 from: debbie@bundlings.com
11. Brensing, K., Linke, K., and Todt, D. (2003) Can dolphins heal by ultrasound? *J. Theor. Biol.* **225(1),** 99–105.
12. Johnston, L. and Bourne, R. (2004) Is There a Dolphin in the House? Retrieved June 21, 2004 from http://healingtherapies.info/dolphin.htm
13. International Dolphin Watch (2006) Can Dolphins Make a Difference? Retrieved May 18, 2006 from: http://www.idw.org/html/dolphin_therapy.html
14. Milne, T. (2003) Dolphins Helped My Child. BBC News Online Staff. Saturday, 22 February, 2003. Retrieved May 14, 2006 from: http://news.bbc.co.uk/1/hi/health/2736863.stm
15. Chess, J. (1997) Stories and Adventures for Zoe. A booklet made by the Chess family for Zoe’s bat-mitzva.
16. Newman, C. and Newman, K. (2004) Amy Swims with Dolphins. *Rett News.* Spring, 17.
17. Berard, G. (1982) *Audition égale comportment*. Maisonneuve, Sainte-Ruffne.
18. Berard, G. (1993) *Hearing Equals Behaviour*. Keats Publishing, New Canaan, CT.
19. Sinha, Y., Silove, N., Wheeler, D., and Williams, K. (2004) Auditory integration training and other sound therapies for autism spectrum disorders. *Cochrane Database Syst. Rev.* **1**. Art. No.: CD003681.pub2. DOI: 10.1002/14651858. CD003681.pub2.
20. Personal communication (IX): Auditory Integration Therapy, received through the Rettnett May 6, 2006 from: annetteb@mymerit.com
21. Fasano, V.A., Nunno, T., Urciolo, R., and Lombard, G. (1964) First observation on the use of oxygen under high pressure for the treatment of traumatic coma. In *Clinical Application of Hyperbaric Oxygen*. Boerema, Brummelkamp, and Meigne, Eds. Elsevier, Amsterdam. pp. 168–173.
22. Muizelaar, J.P. (1989) Cerebral blood flow, cerebral blood volume and cerebral metabolism after severe head injury. In *Textbook of Head Injury.* Becker, D.P. and Gudeman, S.K., Eds. WB Saunders, Philadelphia. pp. 221–240.
23. Ikeda, Y. and Long, D.M. (1990) The molecular basis of brain injury and brain edema: the role of oxygen free radicals. *Neurosurgery* **27(1),** 1–11.

24. Siesjo, B.K., Agardh, C.D., and Bengtsson, F. (1989) Free radicals and brain damage. *Cerebrovasc. Brain Metab. Rev*. **1,** 165–211.
25. Rossignol, D.A. and Rossignol, L.W. (2006) Hyperbaric oxygen therapy may improve symptoms in autistic children. *Med. Hypotheses* **67(2),** 216–228.
26. Golden, Z.L., Neubauer, R., Golden, C.J., Greene, L., Marsh, J., and Mleko, A. (2002) Improvement in cerebral metabolism in chronic brain injury after hyperbaric oxygen therapy. *Int. J. Neurosci.* **112(2),** 119–131.
27. Liptak, G.S. (2005) Complementary and alternative therapies for cerebral palsy. *Ment. Retard. Dev. Disabil. Res. Rev.* **11(2),** 156–163.
28. Waisman, D., Shupak, A., Weisz, G., and Melamed, Y. (1998) Hyperbaric oxygen therapy in the pediatric patient: the experience of the Israel Naval Medical Institute. *Pediatrics* **102(5),** 53–63.
29. Personal communication (X): Hyperbaric Chamber, received through the Rettnett May 2, 2006 from Tom & Heidi Willers: thaiwillers@VERIZON.NET
30. Clark, J.M. (1982) Oxygen toxicity. In *The Physiology and Medicine of Diving*. 3rd ed. Bennett, P.B. and Elliott, D.H., Eds. Bailliere, Tindall and Cox, London. pp. 200–238.
31. Bennett, M.H., Trytko, B., and Jonker, B. (2004) Hyperbaric oxygen therapy for the adjunctive treatment of traumatic brain injury. *Cochrane Database Syst. Rev.* **4**. Art. No.: CD004609.pub2. DOI: 10.1002/14651858.CD004609.pub2.
32. Nuthall, G., Seear, M., Lepawsky, M., Wensley, D., Skippen, P., and Hukin, J. (2000) Hyperbaric oxygen therapy for cerebral palsy: two complications of treatment. *Pediatrics* **106(6),** 80–84.
33. Birch, S., Hesselink, J.K., Jonkman, F.A., Hekker, T.A., and Bos, A. (2004) Clinical research on acupuncture. Part 1. What have reviews of the efficacy and safety of acupuncture told us so far? *J. Altern. Complement. Med.* **10(3),** 468–480.
34. Acupressure Institute home page. Retrieved from: http://www.acupressure.com
35. Berman, B.M. (2001) Clinical applications of acupuncture: an overview of the evidence. *J. Altern. Complement. Med.* **7(Suppl 1),** S111–118.
36. Kulkarni, S., O'Farrell, I., Erasi, M., and Kochar, M.S. (1998) Stress and hypertension. *WMJ* **97(11),** 34–38.
37. Ward, S.L. (1998) Caring and healing in the 21st century. *MCN Am. J. Matern. Child Nurs.* **23(4),** 210–215.
38. Personal communication (II): Acupressure/energy healing/reflexology, received through the Rettnett May 6, 2006 from: annetteb@mymerit.com
39. Ersser, S. (1990) Touch and go. *Nurs. Stand.* **4,** 39.
40. Crawford, N.W., Cincotta, D.R., Lim, A., and Powell, C.V.E. (2006) A cross-sectional survey of complementary and alternative medicine use by children and adolescents attending the University Hospital of Wales. *BMC Complement. Altern. Med.* **6,** 16.
41. Price, S. and Price, L. (1999) *Aromatherapy for Health Professionals*. 2nd ed. Churchill Livingston, London.
42. Smith, C.A., Collins, C.T., Cyna, A.M., and Crowther, C.A. (2003) Complementary and alternative therapies for pain management in labour. *Cochrane Database Syst. Rev.* CD003521.
43. Gedney, J.J., Glover, T.L., and Fillingim, R.B. (2004) Sensory and affective pain discrimination after inhalation of essential oils. *Psychosom. Med.* **66,** 599–606.
44. Leach, M.J. (2004) A critical review of natural therapies in wound management. *Ostomy Wound Manage.* **50,** 36–40.
45. Buckle, J. (1997) *Clinical Aromatherapy in Nursing*. Edward Arnold, London.
46. Motomura, N., Sakurai, A., and Yotsuya, Y. (2001) Reduction of mental stress with lavender odorant. *Percept. Mot. Skills* **93,** 713–718.
47. Goel, N., Kim, H., and Lao, R.P. (2005) An olfactory stimulus modifies nighttime sleep in young men and women. *Chronobiol. Int.* **22(5),** 889–904.
48. Kuriyama, H., Watanabe, S., Nakaya, T., Shigemori, I., Kita, M., Yoshida, N., Masaki, D., Tadai, T., Ozasa, K., Fukui, K., and Imanishi, J. (2005) Immunological and psychological benefits of aromatherapy massage. *Evid. Based Complement. Altern. Med.* **2(2),** 179–184.
49. Price, S. and Price, P.P. (1996) *Aromatherapy for Babies and Children*. Thorsous, London.
50. U.S. Department of Labor. Bureau of Labor Statistics Occupational Outlook Handbook: Chiropractors. Retrieved July 2, 2006 from: http://www.bls.gov/oco/ocos071.htm
51. Personal communication (VI): Chiropractor, received through the Rettnett May 6, 2006 from: annetteb@mymerit.com
52. Craniosacral Therapy. An Internet site. Materials extracted May 17, 2006 from: http://www.eparent.com/welcome/alternative.htm
53. Webhealth. Craniosacral Therapy. Information on the subject of alternative medicine extracted May 18, 2006 from: http://www.webhealth.co.uk/therapies/craniosacral_therapy.asp
54. Personal communication (XI): Cranio-Sacral therapy for Allie, received May 6, 2006 from: spaggis@comcast.net
55. Personal communication (XII): Cranial Sacral therapy, received January 22, 1997 from: ATCath@aol.com
56. Wirth-Pattullo, V. and Hayes, K.W. (1994) Interrater reliability of craniosacral rate measurements and their relationship with subjects' and examiners' heart and respiratory rate measurements. *Phys. Ther.* **74,** 908–916.
57. Wikipedia. Myofascial Release. Information extracted June 27, 2006. from: http://en.wikipedia.org/wiki/Myofascial_Release
58. Personal communication (VIII): Myofascial release, received through the Rettnett May 2, 2006 from: joanchris2000@optonline.net

59. Kovalik, D. (1995) Reiki as an alternative healing method. *Common Factor* **(10),** 9.
60. Nield-Anderson, L. and Ameling, A. (2000) The empowering nature of Reiki as a complementary therapy. *Holist. Nurs. Pract.* **14(3),** 21–29.
61. DiNucci, E.M. (2005) Energy healing: a complementary treatment for orthopaedic and other conditions. *Orthop. Nurs.* **24(4),** 259–269.
62. Personal communication (V): Reiki, received through the Rettnett May 6, 2006 from: annetteb@mymerit.com
63. Spa finder, the global spa resources. Retrieved May 18, 2006 from: http://www.spafinder.com/spalifestyle/spa101/glossary_S.jsp
64. Personal communication (III): Treager massage therapy. A talk with parents. IRSA annual conference, May 1999, Las Vegas, NV.
65. The center for cognitive rehabilitation and neuro-feedback. Information regarding cognitive rehabilitation. Retrieved July 29, 2006 from: http://www.rehabgeorgia.com/cognitive.htm
66. Cicerone, K.D., Dahlberg, C., Kalmar, K., Langenbahn, D.M., Malec, J.F., Bergquist, T.F., Felicetti, T., Giacino, J.T., Harley, J.P., Harrington, D.E., Herzog, J., Kneipp, S., Laatsch, L., and Morse, P.A. (2000) Evidence-based cognitive rehabilitation: recommendations for clinical practice. *Arch. Phys. Med. Rehabil.* **81(12),** 1596–1615.
67. Gillette, Y. and DePompei, R. (2004) The potential of electronic organizers as a tool in the cognitive rehabilitation of young people. *NeuroRehabilitation* **19(3),** 233–243.
68. Personal communication (XV): Cognitive Rehabilitation, received September 26, 1996 from: DentonJB@aol.com.
69. Autism Behavioural Intervention Association. ABA – Background information. Retrieved May 17, 2006 from: http://home.vicnet.net.au/~abia/aba/whatisaba.htm
70. Jones, R.S.P. and McCaughey, R.E. (1992) Gentle teaching and applied behavioral analysis. *J. Appl. Behav. Anal.* **25(4),** 853–867.
71. Steele, D. (1995) Gentle Teaching: A Value Based Framework for Helping Others. A viewpoint document. Retrieved through ERIC data base, May, 2006.
72. Slonims, V. Can you advise me whether ABA is now considered a good treatment for an individual with Rett syndrome? An answer to a question by parents. Retrieved May 13, 2006 from: http://www.rettsyndrome.org.uk/_downloads/Comm_Q5.pdf
73. Kelsey's story. Retrieved June 13, 2006 from: http://www.geocities.com/pflowerett/kelstory.html
74. Sasha Marley Thomas. A personal profile presented through the International Rett Syndrome Association web site. Retrieved May 13, 2006 from: http://www.rettsyndrome.org/content.asp?contentid=935
75. Advanced BioMechanical Rehabilitation (ABR). Information extracted from official web site of method initiators. Retrieved May 6, 2006 from: http://www.blyum.com/
76. Personal communication (I): Advanced Biomechanical Rehabilitation (ABR), received through the Rettnett May 6, 2006 from: thaiwillers@VERIZON.NET
77. Holm, V.A. (1983) A western version of the Doman-Delacato treatment of patterning for developmental disabilities. *West. J. Med.* **139,** 553–556.
78. Sieben, R.L. (1977) Controversial medical treatments of learning disabilities. *Acad. Ther.* **13,** 133–147.
79. American Academy of Pediatrics (1983) The Doman-Delacato treatment of neurologically handicapped children. *Exceptional Parent* **13,** 40–43.
80. American Academy of Pediatrics, Committee on Children With Disabilities (1999) The treatment of neurologically impaired children using patterning. *Pediatrics* **104,** 1149–1151.
81. Personal communication (XIV): Rehabilitation, received January 3, 1997 from: jgeorge@multi-media.ca
82. Personal communication (XIII): Rehabilitation, received January 3, 1997 from: jparris6@netpath.net
83. Anand, B.K. (1991) Yoga and medical sciences. *Indian J. Physiol. Pharmacol.* **35(2),** 84–87.
84. Corby, J.C., Roth, W.T., Zarcone, V.P., Jr., and Kopell, B.S. (1978) Psychophysiologic correlates of the practice of tantric yoga meditation. *Arch. Gen. Psychiatry* **35(5),** 571–577.
85. Ramaratnam, S. and Sridharan, K. (2002) Yoga for epilepsy. *Cochrane Database Syst. Rev*. 1. Art. No.: CD001524. DOI: 10.1002/14651858.CD001524.
86. Panjwani, U., Gupta, H.L., Singh, S.H., Selvamurthy, W., and Rai, U.C. (1995) Effect of Sahaja yoga practice on stress management in patients of epilepsy. *Indian J. Physiol. Pharmacol.* **39(2),** 111–116.
87. Schell, F.J., Allolio, B., and Schonecke, O.W. (1994) Physiological and psychological effects of hatha-yoga exercise in healthy women. *Int. J. Psychosom.* **41(1–4),** 46–52.
88. Neubert, D.A., Moon, M.S., and Grigal, M. (2004) Activities of students with significant disabilities receiving services in postsecondary settings. *Educ. Train. Dev. Disabil.* **39(1),** 16–25.
89. Personal communication (VII) Yoga, received through the Rettnett May 2, 2006 from: sterken1@verizon.net.
